# Motor and Sensory Function as Predictors of MCI and Dementia in the Baltimore Longitudinal Study of Aging (BLSA)

**DOI:** 10.1093/geroni/igab046.1699

**Published:** 2021-12-17

**Authors:** Jennifer Schrack, Amal Wanigatunga, Yurun Cai, Hang Wang, Yuri Agrawal, Susan Resnick, Luigi Ferrucci, Qu Tian

**Affiliations:** 1 Johns Hopkins Bloomberg School of Public Health, Baltimore, Maryland, United States; 2 Johns Hopkins University, Baltimore, Maryland, United States; 3 Otolaryngology, Baltimore, Maryland, United States; 4 National Institute on Aging, Baltimore, Maryland, United States

## Abstract

Motor and sensory impairments are linked with dementia risk, but whether there is a joint effect of deficits in motor and sensory function is unknown. We analyzed 649 BLSA participants (aged 72±11 years; 55% women; 68% white) who had concurrent baseline 6-meter usual gait speed and sensory function (vision, hearing) between 2012-2019. Mild cognitive impairment (MCI) and dementia were adjudicated during an average follow-up of 3 years. We examined the association between baseline gait speed, z-scored sensory function, and a gait*sensory interaction with risk of MCI/dementia using Cox proportional hazard models, adjusted for demographics and chronic conditions. Each .01 m/s faster baseline gait was associated with a reduced risk (HR:0.98 (0.96-0.99)) of MCI/dementia, and each 1 SD higher in hearing and vision z-score was associated with an increased risk (HR:1.84 (1.1-3.1)) increased risk. The was no significant interaction, suggesting motor and sensory impairments may be independently associated with MCI/dementia risk.

